# Distal tibial distraction osteogenesis—an alternative approach to addressing limb length discrepancy with concurrent hindfoot and ankle reconstruction

**DOI:** 10.1186/s13018-019-1264-0

**Published:** 2019-07-30

**Authors:** Todd M. Chappell, Casey C. Ebert, Kevin M. McCann, Byron L. Hutchinson, Edgardo Rodriguez-Collazo

**Affiliations:** 1 Franciscan Foot & Ankle Associates, 1608 S J St., 4th Floor, Tacoma, WA 98405 USA; 2 Department of Veterans Affairs, 2360 E Pershing Blvd, Cheyenne, WY 82001 USA; 3 St. Cloud Orthopedics, 1901 Connecticut Ave South, Sartell, MN 56377 USA; 4 Franciscan Foot & Ankle Institute, 34509 9th Ave S, Ste 306, Federal Way, WA 98003 USA; 5 Department of Surgery, AMITA Health St. Joseph Hospital, Chicago Foot & Ankle Deformity Correction Center, 875 N. Dearborn St. Ste 400, Chicago, IL 60610 USA

**Keywords:** Distraction osteogenesis, Ankle replacement, Avascular necrosis, Talus, Limb length discrepancy, Distal tibial corticotomy

## Abstract

**Background:**

Limb length discrepancy (LLD) in the setting of concurrent hindfoot and ankle deformity poses an added level of complexity to the reconstructive surgeon. Regardless of etiology, a clinically significant LLD poses additional challenges without a forthright and validated solution. The purpose of the current study is to determine whether reconstructive hindfoot and ankle surgery with concurrent lengthening through a distal tibial corticotomy is comparable to other treatment alternatives in the literature.

**Patients and methods:**

A retrospective review of hindfoot and ankle deformity correction utilizing Ilizarov circular external fixation with concurrent distal tibial distraction osteogenesis from July 2009 to September 2014 was conducted.

**Results:**

This study included 19 patients with a mean age of 47.47 ± 13.36 years with a mean follow up of 576.13 ± 341.89 days. The mean preoperative LLD was 2.70 ± 1.22 cm and the mean operatively induced LLD was 2.53 ± 0.59 cm. The mean latency period was 9.33 ± 3.47 days and distraction rate was 0.55 ± 0.16 mm/day. The mean distraction length was 2.14 ± 0.83 cm and mean duration of external fixation was 146.42 ± 58.69 days. The time to union of all hindfoot and ankle fusions was 121.00 ± 25.66 days with an overall fusion rate of 85.71%.

**Conclusions:**

The successful treatment of hindfoot and ankle deformity correction in the setting of LLD using the technique of a distal tibial corticotomy and distraction osteogenesis is reported and illustrates an additional treatment technique with comparable measured outcomes to those previously described. We urge that each patient presentation be evaluated with consideration of all described approaches and associated literature to determine the current best reconstructive approach as future studies may validate or replace the accepted options at present.

## Background

Orthopedic pathology affecting the hindfoot and ankle can be debilitating to the ambulatory patient, especially when accompanied by limb length discrepancy (LLD). Patients with a broad array of diagnoses may be categorized as having hindfoot and ankle deformity with accompanying LLD, these include talar avascular necrosis (AVN), failed total ankle replacement (TAR), congenital or post-traumatic LLD, and those with unintended sequela of surgical interventions or failed hindfoot and ankle reconstructive attempts, e.g., nonunion and/or malunion. Regardless of the etiology, these pathologies involving LLD can lead to functional and anatomical changes that may increase patient morbidity [1–4]. While it has been suggested that a LLD greater than 2–2.5 cm is poorly tolerated [5], a LLD of 0.5–1 cm is perhaps desirable in situations of preexisting or concurrent hindfoot fusions [6]. It has, however, also been reported that as little as a 3-mm LLD can cause postural changes, which over time, may lead to degenerative changes and adaptations of the kinetic chain [7–10]. Deficient limb lengths not amenable to conservative management may require surgical intervention.

A resultant bone deficit and/or LLD has been addressed in multiple ways including fusion of the hindfoot and ankle with accepted loss of length [1, 4, 11], bone grafting procedures of many varieties [12–16], implantable trabecular metal grafts [17–21], distraction osteogenesis [22–25], the use of internal and/or external hardware, and any combination of the above [3, 6, 13, 26–28]. Despite the growing number of treatment alternatives and the various suggested treatment algorithms that are present in the literature, [29] there is no proven gold standard to date.

This study proposes an application of a sparsely published approach to addressing LLD in the distal tibia and ankle. Distal tibial corticotomy (DTC) followed by distraction osteogenesis has been employed to address LLD when faced with complicated ankle fusion. [24] The approach described in this study is similar to the previously reported technique and includes the concomitant arthrodesis of the hindfoot followed by lengthening through a DTC utilizing Ilizarov circular external fixation (ICEF). There is a paucity of reports on distal tibial distraction osteogenesis (DTDO), and as far as the authors are aware, this approach to hindfoot deformity and LLD has not been described in the literature. These surgical techniques have been employed for the past several years to address lower limb deformity correction in the setting of LLD post-reconstruction with the desired outcome of limb salvage and restoration of limb length to prevent the comorbidities associated with limb length inequality.

This approach to treating bone defects of the hindfoot is equivalent to, and in some cases, superior to other treatment alternatives in the literature as it pertains to limb salvage, osseous union, and restoration of limb length. The purpose is to review the outcomes of such reconstructions in terms of radiographic deformity correction and limb length restoration to determine whether or not this technique is comparable to other treatment alternatives in the literature.

## Patients and methods

Following Institutional Review Board (IRB) approval, a retrospective chart review of hindfoot, ankle, and lower limb deformity correction utilizing ICEF with concurrent DTDO from July 2009 to September 2014 was conducted. Current Procedural Terminology (CPT) codes (apply bone fixations device, 20692; osteotomy, tibia, 27705; osteotomy of tibia fibula, 27709; lengthening tibia/fibula, 27715) were searched for patients undergoing reconstructive surgery at our facilities. This search revealed 172 results. Operative reports and clinical and radiographic charts were then reviewed for all cases to included patients who underwent surgical reconstruction of hindfoot and ankle deformity with simultaneous DTDO utilizing ICEF to address LLD as a component of their overall deformity. Patients were excluded if they were less than 18 years of age, had inadequate radiographic imaging, or less than 6 months of follow-up. This review resulted in a total of 19 patients. A clinical and radiographic review of patient records was then conducted to determine demographic data, (Table 1) preoperative diagnosis, concurrent procedures, distraction length, duration of external fixation, minor complications, major complications, union rates, and objective deformity correction outcomes (Table 2).

**Table 1:** Patient demographics

	*n*	%	Range	Mean	SD	Total *n*
Age			43–74	47.47	13.36	19
Sex						19
Male	12	63%				
Female	7	37%				
Laterality						19
Left	4	21%				
Right	15	79%				
BMI			24–38	27.72	4.05	19
Etiology						19
Tibial fracture	7	37%				
Clubfoot deformity	5	26%				
Talar fracture	3	16%				
Malpositioned fusion	2	10.5%				
Gunshot wound	2	10.5%				
Comorbidities						
Low back pain	12	63%				
Hyperlipidemia	4	21%				
HTN	3	16%				
Depression	2	10.5%				
Smoking	2	10.5%				
Reiter’s disease	1	5%				
DM	0	0%				
Follow up (weeks)			33.71–220	77.39	44.37	

*Abbreviations*: *BMI* body mass index, *HTN* hypertension, *DM* diabetes mellitus

**Table 2:** Procedure data

Patient	Preoperative diagnosis	Procedures	Level of corticotomy and correction parameters	Distraction length (cm)	Result of ankle fusion and foot correction^1^	Results of tibial deformity correction^2^	Duration of external fixation (months)	Complications
1	Tibial procurvatum and varum	Tibial osteotomy	Wedged tibial osteotomy at the level of deformity/acute correction	2.45		Acceptable	11.40	Delayed consolidation of regenerate requiring grafting
2	Tibial procurvatum	Tibial osteotomy	Straight tibial osteotomy at the level of deformity/gradual correction	0.65		Excellent	5.42	None
3	Failed total ankle replacement	Total ankle explant, TC fusion, DTC	Distal	1.70	Excellent		6.28	None
4	Tibial procurvatum and varum, post-traumatic ankle arthritis	Tibial osteotomy, TTC fusion	Wedged tibial osteotomy at the level of deformity/acute correction	1.50	Excellent		4.14	Pin site irritation & late regenerate fracture requiring TTC IM rod
5	Tibial varum	Tibial osteotomy	Wedged tibial osteotomy at the level of deformity/acute correction	2.20		Excellent	5.65	None
6	Talar AVN	Talectomy, TC fusion, DTC	Distal	2.00	Poor		4.60	TC infected nonunion
7	Malpositioned TTC fusion	V osteotomy, DTC	Distal	2.10	Good		7.16	None
8	Tibial procurvatum, intrinsic ankle varus, cavovarus foot	Tibial osteotomy, ankle arthrotomy, gastrocnemius recession, Dwyer	Wedged tibial osteotomy at the level of deformity/acute correction	0.90		Acceptable	3.81	Cellulitis
9	Tibial recurvatum	Tibial osteotomy	Wedged tibial osteotomy at the level of deformity/acute correction	2.50		Acceptable	4.50	Neuritis
10	Talar AVN	Talectomy, TC fusion, DTC	Distal	1.50	Excellent		5.32	Delayed consolidation of regenerate
11	Residual clubfoot	V osteotomy, DTC	Distal	3.00		Acceptable	3.22	None
12	Residual clubfoot	V osteotomy, DTC	Distal	2.80		Acceptable	3.22	None
13	Malpositioned hindfoot fusion	Lateral calcaneal slide, DTC	Distal	2.40		Excellent	3.68	None
14	Malpositioned hindfoot fusion, tibial recurvatum, and varum	Tibial osteotomy	Wedged tibial osteotomy at the level of deformity/acute correction	4.00	Good		3.68	Wound dehiscence
15	Intrinsic ankle varus	Lateral calcaneal slide, DTC	Distal	1.40		Excellent	3.68	None
16	Residual clubfoot	V osteotomy, DTC	Distal	1.60		Excellent	3.68	Pin site irritation
17	Talar AVN	Talectomy, TC fusion, DTC	Distal	3.20	Good		3.91	Cellulitis
18	Residual clubfoot	V osteotomy, DTC	Distal	1.80		Excellent	3.45	Wound dehiscence
19	Post-polio equinovarus	V osteotomy, DTC	Distal	3.00		Excellent	4.60	None
Mean ± SD				2.14 ± 0.83			4.81 ± 1.93	

*Abbreviations*: *TC* Tibiocalcaneal, *DTC* distal tibial corticotomy, *TTC* tibiotalocalcaneal, *IM* intramedullary, *AVN* avascular necrosis, *V osteotomy* as described by Kirienko et al. [30], *SD* standard deviation

^1^Post surgical hindfoot and ankle alignment grading as proposed by Katsenis et al. [9]

^2^Post surgical tibial alignment grading as proposed by Schoenleber et al. [2]

Preoperative LLD was evaluated using 51-in. bipedal radiographs and/or scanograms for those patients without suspected intraoperatively induced LLD. For those patients with an operatively induced LLD, defined as LLD due to intraoperative extraction of failed TAR hardware or bone extraction due to AVN, this was calculated based off of intraoperative measurements and postoperative assessment. All cases experienced less than 3 cm of shortening and segmental bone loss due to extraction of pathologic elements and therefore underwent acute shortening intraoperatively. Of note, none of these patients experienced neurovascular compromise.

### Orthobiologics

Bone marrow aspirate concentrate (BMAC) taken from the proximal tibia was used in 9/19 (47.37%) cases to stimulate the fusion site as well as the site of corticotomy. Corticocancellous autograft was used in 3/10 (15.79%) hindfoot and ankle fusion cases consisting of the prepared distal fibular resection combined with a preparation of allogeneic bone matrix containing viable osteogenic cells (Trinity Elite® Orthofix, Inc., Verona, Italy).

### External fixation construct

Following reconstructive procedures of the hindfoot and ankle, application of an ICEF stabilized the correction and was the final step in preparation for the DTC. The construct for the ICEF varied depending on patient differences and pathology addressed; however, it most commonly consisted of a three-ring tibial block spanning the DTC site fixed to the extremity with a combination of 4-mm half pins and tensioned wires in the two proximal tibial rings for biplane stabilization. The distal tibial ring was fixed with two simultaneously tensioned opposing olive wires and this tibial block was connected to a footplate secured to the tibial block with compression rods and fixed to the foot with two simultaneously tensioned opposing olive wires in the calcaneus and two additional wires in the mid to forefoot. The hindfoot fusion sites were compressed as appropriate depending on concurrent procedures.

### Surgical technique

The DTC was completed through an anterior approach at the level of the deformity apex in cases of distal tibial angular deformity. Acute correction at the site of angular tibial deformity was conducted in 6/7 (85.71%) patients via a wedge osteotomy due to its relatively small angular deviation. The remaining case of tibial deformity was corrected gradually due to its high angle of deformity and potential of neurovascular injury with acute correction. In cases with an absence of distal tibial angular deformity, the corticotomy was conducted in similar fashion as close to the distal tibial meta-diaphyseal junction as possible without interfering with fixation elements for stability. Due to initial success lengthening distally at the site of tibial angular deformity, this method was applied to patients who required < 4 cm of lengthening concurrent with their hindfoot and ankle deformity correction. Fixation elements prohibiting DTC as close as possible to the distal tibial meta-diaphyseal junction, and patients requiring > 4 cm of length were not considered candidates for this technique and may be better served by a proximal tibial osteotomy.

The DTC was initiated through the anterior cortex using a drill bit with the use of cold saline irrigation. The DTC was then completed using a series of drill holes connected by sharp osteotomes taking care to not disturb the medullary canal. Once the corticotomy was completed, the ICEF was used to compress the site and intraoperative fluoroscopy was used to verify maintained alignment of the distal extremity. Patients were instructed to remain non-weight bearing and follow up within 5–10 days at which time alignment and pin sites were examined and documentation of the acquired LLD was performed as appropriate.

### Clinical monitoring

Depending upon the concurrent procedures for hindfoot and ankle reconstruction, the LLD, and patient comorbidities, various methods were used to determine the timing of weight bearing and dynamization. Limb length was monitored during treatment by clinical evaluation and radiographic monitoring during distraction until LLD was less than 1 cm. Observation of regenerate and serial radiographs to determine the time to fusion were also collected. Regardless of the variability in each case, the time for consolidation was estimated to be twice the duration of distraction and dynamization was initiated based off of clinical judgment and the appearance of radiographic signs of fusion and consolidation of the regenerate.

### Objective radiographic parameters

Objective measures of osseous correction were evaluated using previously published parameters for distal tibial [2], hindfoot and ankle [9], deformity correction including union, presence or absence of infection, position of fusion, and residual LLD post lengthening with less than 1.5 cm being excellent. The goals for position of fusion of the hindfoot and ankle were deemed excellent when the hindfoot was in neutral to slight calcaneus, neutral to 5° of valgus, neutral to slight posterior translation, and 0–15° of external rotation [9]. In situations of tibial deformity alone, the same parameters were evaluated in terms of union, absence or presence of infection, and residual LLD post lengthening according to Schoenleber et al. [2]. When both coronal and sagittal planes were less than 5° from the normal anatomic axis of the tibia, the correction was deemed excellent; however, the position of the hindfoot was not assessed in his criteria [2] which is the rationale for using the combined criteria for objective radiographic evaluation of osseous outcomes (Table 3).

**Table 3:** Objective measures of osseous correction

	Excellent	Good	Fair	Poor
Union	Solid			Nonunion
Infection	Absence			Presence
Deformity	Neutral to slight calcaneus Neutral to 5° valgus External rotation 0–15° Neutral to slight posterior translation	Slight equinus < 5° 5–10° valgus or varus < 5° internal rotation < 1 cm anterior translation	5–10° dorsiflexion or plantarflexion 5° varus or > 10° valgus < 5° internal rotation > 1 cm anterior translation	Worse than before intervention
Hindfoot^1^				
Tibial^2^	Both coronal and sagittal planes within 5° of normal	Acceptable		Neither coronal nor sagittal planes within 5° of normal
		Either coronal or sagittal plane within 5° of normal		
LLD	< 1.5 cm	< 3 cm	> 3 cm	

*Abbreviations*: *LLD* limb length discrepancy

^1^Post surgical hindfoot and ankle alignment grading as proposed by Katsenis et al. [9]

^2^Post surgical tibial alignment grading as proposed by Schoenleber et al. [2]

Means and standard deviations were performed for all parameters using Microsoft Excel (Palo Alto, CA, USA). Outcome comparison statistics were not evaluated due to the small sample size of the hindfoot and tibial deformity groups as well as the variability of the concurrent hindfoot and ankle procedures making comparison between groups and cases challenging.

## Results

**Fig. 1:**
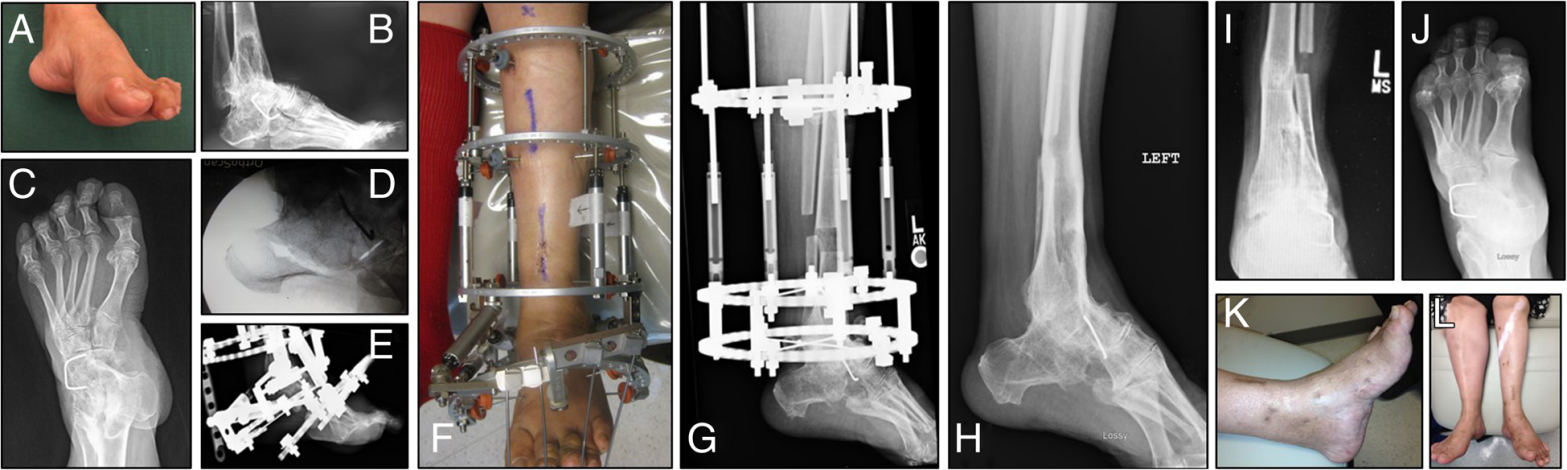
Case #7. Fifty-eight-year-old F with history of clubfoot correction as a teenager. **a** Preoperative appearance of foot. **b**, **c** Preoperative radiographs of cavoadductovarus foot with evidence of prior surgery. **d** Intraoperative fluoroscopic image of V osteotomy. **e**–**g** Clinical and radiographic appearance of frame construct for gradual foot correction and DTC demonstrating regenerate formation. **h**–**j** Postoperative anteroposterior and lateral radiographs demonstrating foot correction and consolidated distal tibial corticotomy site. **k**, **l** Postoperative appearance of the foot and corrected LLD

**Fig. 2:**
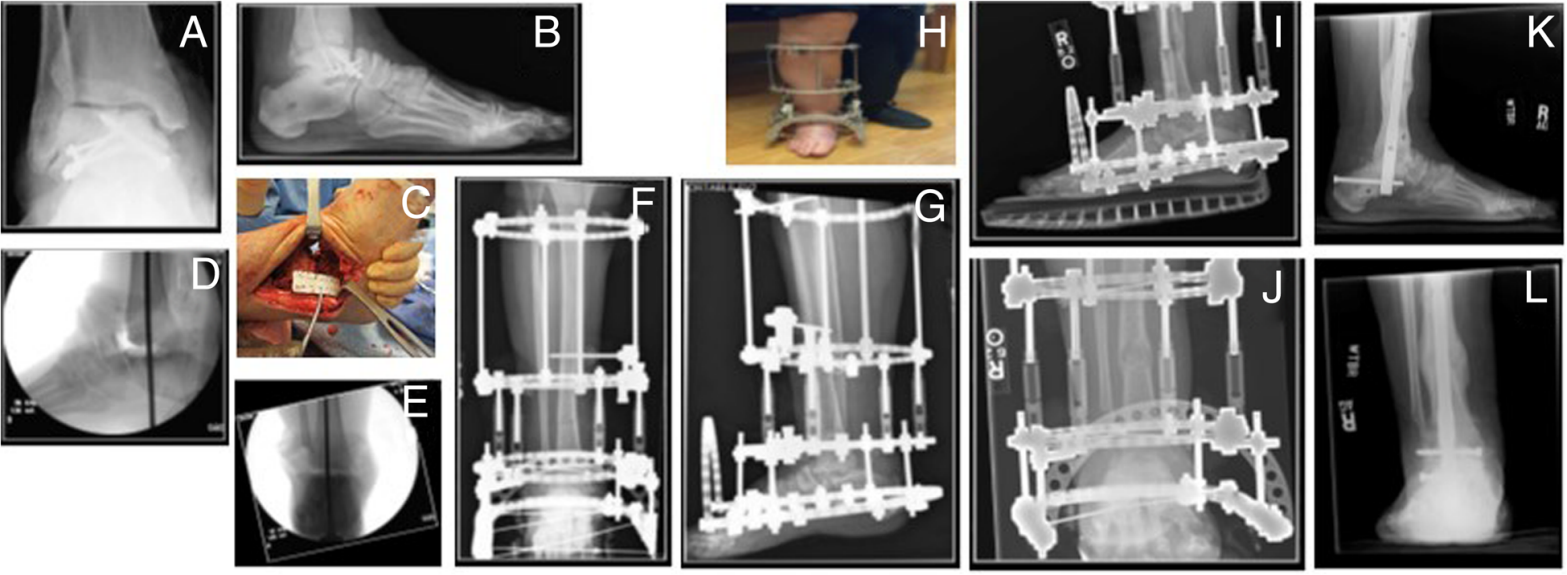
Case #10. Seventy-four-year-old F 9 months s/p motor vehicle accident with closed talus fracture. **a**, **b** Preoperative radiographs demonstrating talar collapse and AVN. **c** Intraoperative image post talectomy. **d**, **e** Intraoperative fluoroscopy of tibiocalcaneal fusion site alignment. **f**, **g** AP and lateral radiographs demonstrating frame construct for tibiocalcaneal fusion and DTC. **h**–**j** Clinical and radiographic appearance of frame construct demonstrating regenerate formation. **k**, **l** Final postoperative AP and lateral radiographs following distal tibial lengthening, tibiocalcaneal fusion, and intramedullary nailing

This study included 12 males and 7 females with a mean age of 47.47 ± 13.36 years (range 22 to 74 years) with a mean body mass index (BMI) of 27.72 ± 4.05. The mean follow-up was 576.13 ± 341.89 days (range 236–1667 days). The most common cause of deformity was tibial fracture 7/19 (36.84%) with 2/7 (28.57%) being open at the time of initial injury. The second most common cause of deformity was history of clubfoot deformity with a remote history of correction 5/19 (26.32%) (Fig. 1) followed by talar fracture 3/19 (15.79%) (Fig. 2) malpositioned hindfoot fusions, and gunshot wounds with 2/19 (10.53%) each. Although the abovementioned diagnoses were the initial cause of deformity, 16/19 (84.21%) had undergone at least one surgery prior to their presentation. The most surgical procedures undergone by a single patient was five.

The mean preoperative LLD was 2.70 ± 1.22 cm and the mean operatively induced LLD was 2.53 ± 0.59 cm. The most common procedure to correct deformity of the hindfoot and ankle was a V osteotomy as described by Kirienko et al. [30] 5/9 (55.56%) followed by tibiocalcaneal fusion 4/9 (44.44%) following explant of TAR or talectomy for AVN. The mean latency period was 9.33 ± 3.47 days. The mean distraction length was 2.14 ± 0.83 cm and mean duration of external fixation was 146.42 ± 58.69 days.

The time to union of hindfoot/ankle fusions was 121.00 ± 25.66 days as determined radiographically by routine postoperative radiographs demonstrating bridging trabeculation of the fusion site and also clinically by the sustained absence of pain and swelling at the surgical site. The overall fusion rate was 6/7 (85.71%). Following the parameters outlined by Schoenleber et al. for tibial deformity correction and Katsenis et al. for hindfoot and ankle deformity correction, excellent union results in 6/7 (85.71%) patients, excellent infection results in 18/19 (94.74%) patients, excellent postoperative LLD results in 19/19 (100%) patients, excellent hindfoot and ankle deformity correction results in 4/7 (57.14%) patients, and excellent tibial alignment results in 7/12 (58.33%) patients were achieved (Table 4).

**Table 4:** Osseous correction outcomes

		Excellent	Good	Fair	Poor
Union*		6			1
Infection		18			1
Deformity					
Hindfoot	*n* = 7	4	3		
			Acceptable		
Tibial	*n* = 12	7	5		
LLD		19			
Total		19			

*Abbreviations*: *LLD* limb length discrepancy

*Patients who underwent hindfoot/ankle fusion

Complications were evaluated and categorized as major and minor. Major complications required surgical intervention for resolution whereas minor complications were addressed non-surgically. There were four major complications and seven minor complications. Major complications included delayed consolidation of regenerate in 2/19 (10.53%), late fracture of regenerate in 1/19 (5.26%), and infected nonunion of the tibiocalcaneal fusion site in 1/19 (5.26%). The delayed consolidation was treated with intramedullary (IM) nailing and bone marrow stimulation consisting of autologous BMAC in 1/2 (50%) and the other with bone grafting and internal plate fixation. Both procedures resulted in successful healing. The late fracture of regenerate was treated with tibiotalocalcaneal (TTC) fusion using IM nailing and resulted in a successful outcome. The infected nonunion was treated by debridement, an extended course of intravenous (IV) antibiotics, and definitive placement of an antibiotic cement spacer. This patient is currently ambulating with the assistance of a leg brace (Exosym™ Hanger, Inc., TX, USA) which offloads the extremity and there are currently no plans of revision surgery. Of the seven minor complications, cellulitis, pin site infection, and wound dehiscence were the most common occurring in 2/19 (10.53%) patients each. Neuritis was the second most common minor complication affecting 1/19 (5.26%) patients. Minor complications of an infectious nature were all treated with oral antibiotics to full resolution.

## Discussion

Hindfoot and ankle deformity is a complex pathology and a challenge for the reconstructive surgeon; this complexity is increased in the setting of LLD. The etiology of LLD is varied and often is a result of trauma or prior surgical interventions and/or failures of such interventions. Regardless of the cause of the LLD, there is no consensus as to the most successful method of treatment and several approaches have demonstrated success.

Based off of our experience with distal tibial lengthening, we propose an additional alternative to the previously described methods. The time to union in this study was 121.00 ± 25.66 days with an overall fusion rate of 85.71%. The mean distraction length was 2.14 ± 0.83 cm and mean duration of external fixation was 146.42 ± 58.69 days resulting in an external fixation index of 68.42 days/cm. These results are comparable to other described methods in the literature making this technique a successful alternative when used by surgeons with appropriate experience and technical expertise.

The consistent search for alternate treatment options for this challenging pathology has resulted in multiple studies reporting on various methods to achieve successful hindfoot and ankle fusions in the setting of LLD. In a review of published studies, it was suggested that the most straightforward method of treatment is direct fusion with loss of length when the resultant LLD is < 2 cm and acceptable to the patient [11]. A similar conclusion was made in a study reviewing cases of talar AVN which demonstrated that successful tibiocalcaneal fusion can be achieved without the use of structural bone graft [4]. Fragomen et al. reported on complex ankle fusion in 91 patients utilizing the Ilizarov method and noted greater LLD was associated with a higher risk of nonunion although the majority of their patients did not undergo lengthening to normalize limb lengths. They recommended that patients with a LLD > 2.5 cm and < 70 years old be considered candidates for lengthening. Their results indicate that the 24/91 (26.37%) patients who underwent lengthening resulted with an 83% rate of union [1].

Although it has been shown that correction of LLD is not necessary to obtain successful fusion or positive patient outcomes, the majority of the current literature aims for correction of LLD. Some of the more recent literature has been directed toward implantation of metal spacers in an attempt to limit the post treatment LLD. There has been a myriad of reportedly successful implantations; however, they are commonly single case reports or small case series. In 2008, the use of Harms cages was reported in three patients after failed TAR. All required revision surgery with IM retrograde nail fixation after removal of the Harms cages, and 2/3 (66.66%) successfully fused the second attempt [17]. The use of a titanium cage with morselized cancellous graft was also reported; however, retrograde IM nail fixation was added for stability and successful results were reported in two patients [19]. Sagherian et al. has reported on the successful use of porous tantalum metal spacers in ankle and hindfoot surgery as well as various other foot pathologies with good results reporting 100% fusion rate of the implant-bone interface. Fusion was defined as an absence of lucency at the bone-tantalum interface on plain radiography and maintenance of correction with the clinical absence of pain, tenderness or swelling [18, 21]. Although there may be some potential for successful implementation of these devices in the future, it would require increased patient numbers and sustained evidence of successful incorporation into bone for widespread adoption.

One of the most widely reported methods for addressing hindfoot and ankle deformity in the setting of LLD has been the use of structural bone grafting techniques. These have included tricortical iliac crest autograft, fresh-frozen femoral head allograft (FFFHA), free vascularized bone autografts, and fibular strut autografts to name a few. In 2009, the use of an anterior double plating system for tibiotalar arthrodesis was reported on 29 patients. Amongst this group, 9 patients had failed TAR for which they utilized FFFHA. Although the mean follow up for this study was 43.9 months, it appears that they elected not to comment on whether or not these grafts underwent late collapse, it was reported that they had a 100% union rate with no evidence of collapse during the first 12 months. They did comment however that they could not conclude that such grafts will not collapse in the future [15].

Others have reported on their experience with FFFHA in patients treated for failed TAR. In a review of 9 patients treated with FFFHA and internal fixation, it was reported as a 55.6% union rate with 4/9 patients failing at the graft subtalar joint (STJ) interface. They believed that it was their selected difficult approach to preparation of the STJ through the anterior incision to blame for their poor result and therefore their revision cases were approached through a sinus tarsi incision and eventually healed the remainder of the patients. They also reported a late fracture of the FFFHA [12]. In 2013, Jeng et al. reported on the use of FFFHA for TTC fusion in 32 patients. The average size of the FFFHA implanted was 38 mm, and IM rod fixation was the primary method of choice although there were 6 patients that received internal plate fixation. Only 50% of these grafts went on to heal and the average collapse of the FFFHA at final follow up was measured at 3.6 mm [14]. A similar study in 2014 utilized FFFHA and a combination of internal plate and rod fixation in 17 patients for failed TAR. Their results are superior to Jeng et al. with a 76.5% union rate initially at 3.7 months and 3/4 healed after a second attempt at fusion [13].

In a deviation from the use of the traditional FFFHA in the treatment of failed TAR and talar AVN with bone void, another group utilized autogenous fibula and IM nailing with an anterior plate in 6 patients. Their technique involved resection of the distal fibula and subsequent splitting into medial, lateral, anterior, and posterior quarters of sufficient length to fill the bony void. These segments were placed circumferentially around the intramedullary (IM) nail and although patient numbers are low, they achieved a 100% union rate at final follow up (26 months) [16]. It is obvious that there remains room for improvement in these difficult cases. The above reports are examples of the expectations with regard to the use of structural bone grafting techniques and internal fixation to preserve limb length.

The technique of external fixation provides a myriad of options in the treatment of skeletal defects and these applications have been employed in the treatment of foot and ankle deformity correction and segmental defects. In a direct comparison of bone grafting versus distraction osteogenesis, Green et al. reported similar outcomes between the two groups. The average defect treated by Papineau grafting was 4 cm as compared to the average defect treated by bone transport of 5 cm. They compared the duration of external fixation and this resulted in equivalency at 1.9 months/cm. The outcomes revealed that 2/15 (13.33%) in the Papineau grafting required additional procedures for nonunion as compared to 7/17 (41.18%) in the distraction osteogenesis group with all of these affecting the docking site [22]. More recently, 11 talectomy and TTC fusion procedures were reviewed with concurrent proximal tibial lengthening in 8/11 patients. An 81.82% union of the fusion site was achieved and a mean length of 4 cm over 7 months in external fixation [23]. McCoy et al. reviewed 7 patients with failed TAR with an average follow up of 58 months. Due to the loss of length after implant removal and residual talus debridement, four patients elected to undergo proximal tibial lengthening. These patients were lengthened an average of 4.6 cm and resulted with an external fixation index of 42.6 days/cm with 100% union of the distal fusion site [6]. These studies all point to the success of proximal tibial lengthening to address LLD when faced with distal tibial and hindfoot pathology.

Similar to the approach reported in this current study, Sakurakichi et al. performed ankle fusion and tibial lengthening through a distal corticotomy. Their review consisted of six patients separated into two distinct treatment groups based on defect size. Three patients underwent distal corticotomy for defects < 3 cm and the remaining three underwent bone transport for defects > 5 cm. The average length obtained in the compression distraction group was 1.93 cm with an external fixation index of 144 days/cm while the bone transport group was 4.1 cm and an external fixation index of 90 days/cm [24]. Similar to this study, we do not recommend this approach for defects > 3–4 cm, but it does demonstrate the ability to lengthen through a DTC to obtain successful fusion and deformity correction of complicated hindfoot and ankle deformity with LLD.

As evidenced in the literature, there have been many approaches to the treatment of hindfoot and ankle pathology in the setting of LLD, whether that be operatively induced or present preoperatively. The literature also demonstrates that there is no gold standard approach to treatment and further confirms the limb-threatening challenge that this group of pathologies presents to the reconstructive surgeon. The consecutive case selection of the current study and evaluation of deformity correction outcomes based off of previously reported methods of osseous correction is a strength of the study.

Although the authors of the current study have reported a successful method for treatment, there remain limitations to this study. Due to the infrequency of this presenting pathology, the authors reviewed patients treated at two different sites by two different surgeons, and although the treatment algorithm is uniform, there are minor differences in technique that cannot be ignored. However, the success of treatment evidenced by these results indicates the reproducibility of this technique. It would have been ideal to increase the minimum follow up time of the patients; however, doing so likely would not have altered the radiographic measurements reported. Finally, there was a lack of patient function and satisfaction scoring due to the retrospective nature of this study design. A proposal for further works describing and comparing reconstructive techniques with both subjective and objective measures for these unique and challenging presentations is made in an effort to allow for better understanding and reproducibility of the most appropriate surgical approaches and their outcomes.

## Conclusions

In conclusion, the authors report the successful treatment of hindfoot and ankle deformity correction in the setting of LLD using the technique of a DTC and distraction osteogenesis. Although latency period and distraction rate are variable and unique to each patient, the authors experience indicates that a latency period of 7–10 days and a distraction rate of < 1 mm/day improves regenerate formation in the distal tibia. While the strength of the current study is not sufficient to recommend this technique for all patients presenting with the indications for this procedure, it does provide an additional alternative for these challenging deformities.

## Data Availability

The datasets generated and/or analyzed during the current study are not publicly available due to sensitivity of patient information contained in the data sets but portions are available from the corresponding author on reasonable request.

## Abbreviations

AVN: Avascular necrosis
BMAC: Bone marrow aspirate concentrate
BMI: Body mass index
CPT: Current procedural terminology
DTC: Distal tibial corticotomy
DTDO: Distal tibial distraction osteogenesis
FFFHA: Fresh-frozen femoral head allograft
ICEF: Ilizarov circular external fixation
IM: Intramedullary
LLD: Limb length discrepancy
STJ: Subtalar joint
TAR: Total ankle replacement
